# The International Medical Congress

**Published:** 1890-03

**Authors:** 


### The Tenth International Medical Congress.

In accordance with the decision of the Ninth Congress at Washington, the Tenth International Medical Congress will be held at Berlin from the 4th to the 9th of August, 1890.

By the delegates of the German Medical Faculties and the chief Medical Societies of the German Empire, the undersigned have been appointed members of the General Committee of Organisation. A Special Committee of Organisation has also been appointed for each of the different sections, to arrange the scientific problems to be discussed at the meetings of the respective sections. An International Medical and Scientific Exhibition will also be held by the Congress.

We have the honour to inform you of the above decisions, and at the same time cordially to invite your attendance at the Congress. We should esteem it a favour if you would kindly extend this invitation to your friends in medical circles as way may offer.—Dr. Rudolf Virchow, President; Dr. Von Bergmann, Dr. Leyden, Dr. Waldeyer, Vice-Presidents; Dr. Lassar, Secretary-General.

### *Regulations and Programme.*

I. The Tenth International Medical Congress will be opened in Berlin on Monday, August 4th, 1890, and will be closed on Saturday, August 9th.

II. The Congress shall consist of legally-qualified medical men who have inscribed themselves as members, and have paid for their card of membership. Other men of science who interest themselves in the work of the Congress may be admitted as extraordinary members.

Those who take part in the Congress shall pay a subscription of twenty marks (one pound stg. or $ 5) on being enrolled as members. For this sum they shall receive a copy of the Transactions, as soon as they appear. The enrolment shall take place at the beginning of the Congress. Gentlemen may, however, be enrolled as members by sending the amount of the subscription to the treasurer,* with their name, professional status, and residence appended.

III. The object of the Congress is an exclusively scientific one.

IV. The work of the Congress will be discharged by eighteen different sections.† The members shall declare upon enrolment to which section or sections they intend more particularly to attach themselves.

V. The Committee of Organisation shall, at the opening sitting of the Congress, suggest the election of a definite Committee (or Bureau), which shall consist of a President, three Vice-Presidents, and of a number—as yet undetermined—of Honorary Presidents and Secretaries.

At the first meeting of each section, a President and certain number of Hon. Presidents shall be elected; these

* Treasurer's address: Dr. M. BARTELS, Berlin, S.W., Leipzigerstrasse 75. Please to enclose a visiting-card.

† *SPECIAL SECTIONS.*

1. ANATOMY.
2. PHYSIOLOGY AND PHYSIOLOGICAL CHEMISTRY.
3. GENERAL PATHOLOGY AND PATHOLOGICAL ANATOMY.
4. PHARMACOLOGY.
5. INTERNAL MEDICINE.
6. DISEASES OF CHILDREN.
7. SURGERY.
8. OBSTETRICS & GYNÆCOLOGY.
9. NEUROLOGY & PSYCHIATRY.
10. OPHTHALMOLOGY.
11. OTOLOGY.
12. LARYNGOLOGY & RHINOLOGY.
13. DERMATOLOGY AND SYPHILIGRAPHY.
14. DISEASES OF THE TEETH.
15. HYGIENE.
16. MEDICAL GEOGRAPHY AND CLIMATOLOGY (HISTORY AND STATISTICS).
17. STATE MEDICINE.
18. MILITARY HYGIENE.

latter shall conduct the business of the sections in turn with the Presidents.

On account of the different languages employed, a suitable number of secretaries shall be chosen from among the foreign members. The duties of the foreign secretaries shall be confined to the sittings of the Congress.

After the termination of the Congress, the editing of the Transactions shall be carried out by a committee, specially appointed for this purpose.

VI. The Congress will assemble daily, either for a general meeting or for the labours of the different sections.

The general meetings will be held between eleven and two o'clock. Three such meetings will take place.

The time for the sittings of the various sections will be fixed by the Special Committee of each section, it being understood, however, that no such sittings are to take place during the hours allotted to the general meetings.

Joint sittings of two or more sections may be held, provided that the Bureau of the Congress can offer suitable rooms for such sittings.

VII. The general meetings shall be devoted to

(*a*) Transactions connected with the work and general management of the Congress.

(*b*) Speeches and communications of general interest.

VIII. Addresses in the general sittings, as well as in any extraordinary meetings which may be determined upon, can only be given by those who have been specially requested by the Committee of Organisation.

Proposals relative to the future management of the Congress must be announced to the Committee of Organisation before July 1st, 1890. The committee shall decide whether these proposals are suitable to be introduced for discussion.

IX. In the sittings of the sections, questions and problems will be discussed, which have been agreed upon by the special Committees of Organisation. The communications of those appointed by the committee to report on a subject shall form the basis of discussion. As far as time allows, other communications or proposals, proceeding from members and sanctioned by the Committee of Organisation, may also be introduced for discussion. The Bureau of each section decides as to the acceptance of such offered communications, and as to the order in which they shall come before the meeting, always provided that this point has not been already determined in the sitting itself by a decree of the section.

Scientific questions shall not be put to the vote.

X. Introductory addresses in the sections must, as a rule, not exceed *twenty minutes in length*. In the discussions no more than *ten minutes* are allowed to each speaker.

XI. All addresses and papers in the general and sectional meetings must be handed over to the secretaries, in writing, before the end of the sitting. The Editorial Committee shall decide whether, and to what extent, the written contributions shall be included in the printed Transactions of the Congress. The members who have taken part in the discussions will be requested to hand over to the secretaries before the end of the day, in writing, the substance of their remarks.

XII. The official languages of all the sittings shall be German, English, and French. The regulations, the programme, and the agenda for the day will be printed in all three languages.

It will, however, be allowable to make use of other languages than the above for brief remarks, always provided that one of the members present is ready to translate the gist of such remarks into one of the official languages.

XIII. The acting-president shall conduct the business of each meeting according to the parliamentary rules generally accepted in deliberative assemblies.

XIV. Medical students and other persons, ladies and gentlemen, who are not physicians, but who take a special interest in the work of a particular sitting, may be invited by the President, or be allowed to attend the sitting by special permission.

XV. Communications or enquiries regarding the business of separate sections must be addressed to the managing members thereof. All other communications and enquiries must be directed to the General Secretary, Dr. Lassar, Berlin, N.W., 19 Karlstrasse.

In connection with the Congress, there will be an International Medico-Scientific Exhibition. We therefore cordially invite all who may wish to exhibit or participate in the above exhibition. All exhibits, however, to be of a scientific nature.

The exhibits expected will be as follows :

1. New or improved scientific instruments for biological and special medical purposes, including apparatus for photography and spectral analysis pertaining to medicine.
2. New pharmacological chemical substances and preparations.

3. New pharmaceutical substances and preparations.
4. New food preparations.
5. New or improved instruments for internal and external medicine and allied specialities, including electrotherapy.
6. Plans and models (new) of hospitals; houses for reconvalescents, disinfection, and general bath-houses.
7. New appliances, such as pertain to nursing the sick, including the methods of transportation, and baths for the sick.
8. Apparatus (new) for hygienic purposes.

For applications for exhibits, and information, please address Dr. O. Lassar, Secretary-General, Bureau of the Tenth International Medical Congress, Berlin, N.W., Carlstrasse, No. 19.

Please designate all mail matter relating to the exhibition "Exhibition Affairs," and also enclose a visiting-card or card of the firm, on which the name and residence is plainly written or printed.

The Bureau is open for the present from five to seven p.m.

